# Conventionally instrumented inverse kinematic alignment for total knee arthroplasty: How is it done?

**DOI:** 10.1002/jeo2.12055

**Published:** 2024-06-12

**Authors:** Shane P. Russell, Sara Keyes, Michael T. Hirschmann, James A. Harty

**Affiliations:** ^1^ Department of Orthopaedic Surgery South Infirmary Victoria University Hospital Cork Ireland; ^2^ Department of Orthopaedic Surgery Bon Secours Hospital Cork Cork Ireland; ^3^ Department of Orthopaedic Surgery University College Cork Cork Ireland; ^4^ Department of Orthopaedic Surgery Royal College of Surgeons in Ireland Dublin Ireland; ^5^ Department of Orthopedic Surgery and Traumatology Kantonsspital Baselland Bruderholz Switzerland; ^6^ Department of Clinical Research, Research Group Michael T. Hirschmann, Regenerative Medicine & Biomechanics University of Basel Basel Switzerland

**Keywords:** conventional instrumentation, inverse kinematic alignment, kinematic alignment, mechanical alignment, total knee arthroplasty

## Abstract

**Purpose:**

For primary total knee arthroplasty (TKA), there is an increasing trend towards patient‐specific alignment strategies such as kinematic alignment (KA) and inverse kinematic alignment (iKA), which by restoring native joint mechanics may yield higher patient satisfaction rates. Second, the most recent Australian joint registry report describes favourable revision rates for conventionally instrumented TKA compared to technology‐assisted techniques such as those using navigation, robotics or custom‐cutting blocks. The aim of this technique article is to describe in detail a surgical technique for TKA that: (1) utilises the principles of iKA and (2) uses conventionally instrumented guided resections thereby avoiding the use of navigation, robotics or custom blocks.

**Methods:**

A TKA technique is described, whereby inverse kinematic principles are utilised and patient‐specific alignment is achieved. Additionally, the patellofemoral compartment of the knee is restored to the native patellofemoral joint line. The sequenced technical note provided may be utilised for cemented or cementless components; cruciate retaining or sacrificing designs and for fixed or rotating platforms.

**Results:**

An uncomplicated, robust and reproducible technique for TKA is described.

**Discussion:**

Knee arthroplasty surgeons may wish to harness the emerging benefits of both a conventionally instrumented technique and a patient‐specific alignment strategy.

**Level of Evidence:**

Level V.

**Abbreviations**
3D: three dimensional
ACL: anterior cruciate ligament
AOANJRR: Australian Orthopaedic Association National Joint Replacement Registry
ASA: American Society of Anesthesiologists
A/P: anterior/posterior
BMI: body mass index
CPAK: coronal plane alignment of knee
CR: cruciate retaining
CT: computed tomography
FB: fixed bearing
ICPD: intercompartmental pressure differences
IDI: image derived instrumentation
iKA: inverse kinematic alignment
JLO: joint line obliquity
KA: kinematic alignment
LCS: low contact stress
MA: mechanical alignment
mHKA: mechanical hip knee ankle angle
MPTA: medial proximal tibial angle
PROMS: patient reported outcome measures
PS: posterior stabilised
RP: rotating platform
TKA: total knee arthroplasty

## INTRODUCTION

When adjusted for age, gender, American Society of Anesthesiologists score, Body Mass Index, bearing surface, patellar component usage and stability there is no difference in the rate of revision between total knee arthroplasty (TKA) using navigation or robotic assistance and TKA without using such technologies [43]. Furthermore, according to the 2023 Australian Orthopaedic Association National Joint Replacement Registry (AOANJRR), revision rates for procedures using image‐derived instrumentation or custom‐made cutting blocks, are higher when compared to TKA without technology assistance [43]. While improved accuracy of cuts using robotic assistance has been demonstrated by some studies, multiple randomised studies now demonstrate no long‐term clinical benefit for robotic‐assisted TKA [4, 9, 26, 31, 32, 42].

Technology‐assisted TKA has increased; now accounting for 65.8% of primary TKAs in the AOANJRR [43]. In addition to the superior survivorship now demonstrated for the first time by this registry, conventionally instrumented techniques for TKA may be performed under reduced cost and shorter operating time while avoiding complications such as pin site infection and surgeon learning curves [22, 51].

To combat the 10%–20% of patients that remain dissatisfied following TKA, surgeons have sought to improve outcomes by capitalising on advances in perioperative anaesthesia, rehabilitation protocols, implant design, implant fixation, surgical technique and surgical alignment strategies [5, 41, 48]. Several contemporary instrumented TKA techniques and alignment strategies have been described, with a modern trend away from mechanical alignment (MA) towards personalised alignment strategies such as restricted kinematic alignment (KA) or inverse kinematic alignment (iKA) [48, 49, 50].

Advances in navigation and robotic instrumentation have recently resulted in an abundance of three‐dimensional implant positioning data. Such technologies propose increased surgical precision for implant positioning and fewer alignment outliers [6, 44]. The optimal target for implant positioning remains poorly understood despite such advances. While convincing superior clinical outcomes using technology‐assisted instrumentation is yet to be demonstrated, such technologies do provide important data that may help answer the ideal implant position question [19].

Both MA and KA may be achieved through conventionally instrumented surgical techniques or through technology‐assisted techniques.

The 1973 gold‐standard technique, MA, aims for a straight knee by performing femoral and tibial bony resections perpendicular to the mechanical axis of the lower limb [14, 29, 48]. The knee is then balanced, independent of knee phenotype, by sequential release of the soft tissue structures about the knee, resulting in less favourable patient outcomes [29, 46]. Providing patients with a neutral mechanical hip knee angle (mHKA) or ‘straight leg’, proposed advantages of the ‘one‐alignment‐fits‐all’ MA approach include the technical ease of perpendicular osteotomies, reduced shear forces at the bearing surface and symmetrical medial and lateral compartment loading; all thereby thought to maximise prosthesis survivorship [29]. However, both the hypothesised reduced shear forces and increased survivorship of MA knees now seem to be dispelled [12, 20, 22, 23, 29, 34, 36].

MA fails to account for the significant variability in constitutional lower limb alignment [3, 7, 17]. The utilisation of a general‐purpose, single‐target alignment strategy for all patients (despite historically imperfect outcomes, advances in implant design and understandings of knee motion) is being challenged [2, 25].

The classification of constitutional alignment to either varus, neutral or valgus is oversimplified, for both healthy and arthritic knees and so variations of normal lower limb alignment phenotypes should be considered [15, 16, 18]. Hirschmann et al. observed 43 functional knee phenotypes [17]. For the most common knee phenotypes, MA strategies resulted in significantly more bone resection [25, 39]. To minimise bony resection and preserve the soft tissue envelope of the knee, preoperative planning with a comprehensive understanding of the knee phenotype is necessary [25].

KA strategies restore constitutional knee mechanics by restoring the three kinematic axes of normal knee motion and therefore the prearthritic lower limb alignment [20, 21, 22, 29]. Through a femur‐first philosophy, osteotomies are performed so that the position of the prosthesis replicates prearthritic joint geometry, negating the need for soft tissue releases (a pure measured resection technique). The patient's constitutional mHKA and joint line obliquity (JLO) are thereby preserved. Contrary to MA doctrine, kinematic TKAs demonstrate lower intercompartmental pressure differences, reduce the need for intraoperative bony recuts and reduce the incidence of tibiofemoral lift‐off (due to an overtight contralateral compartment) [28, 29]. KA techniques have demonstrated superior range of motion and superior patient‐reported outcome measures (PROMS) compared to MA techniques in short‐term follow‐up [11, 12].

iKA utilises a tibia‐first philosophy to achieve patient‐specific alignment. Conversely to KA, the tibial resection determines the femoral resections and joint balance. In keeping with kinematic principles, the soft tissue envelope is preserved and balance is achieved through osteotomies that replicate native joint motion and restore native limb alignment [48]. Advantages of iKA include the ability to avoid tibial over‐resection and for independently measured, kinematic resections of both the tibia and femur to be performed [30].

Building on the previously described principles of iKA, a TKA technique is described that utilises the principles of both kinematic femoral and kinematic tibial measured resection principles. In addition, restoration of the third compartment of the knee is kinematically performed during patellofemoral resurfacing. Encouraged by the latest AOANJRR results and reported clinical outcomes, the aim of this paper is to describe in detail a conventionally instrumented iKA technique [24].

## METHODS

**Figure 1:**
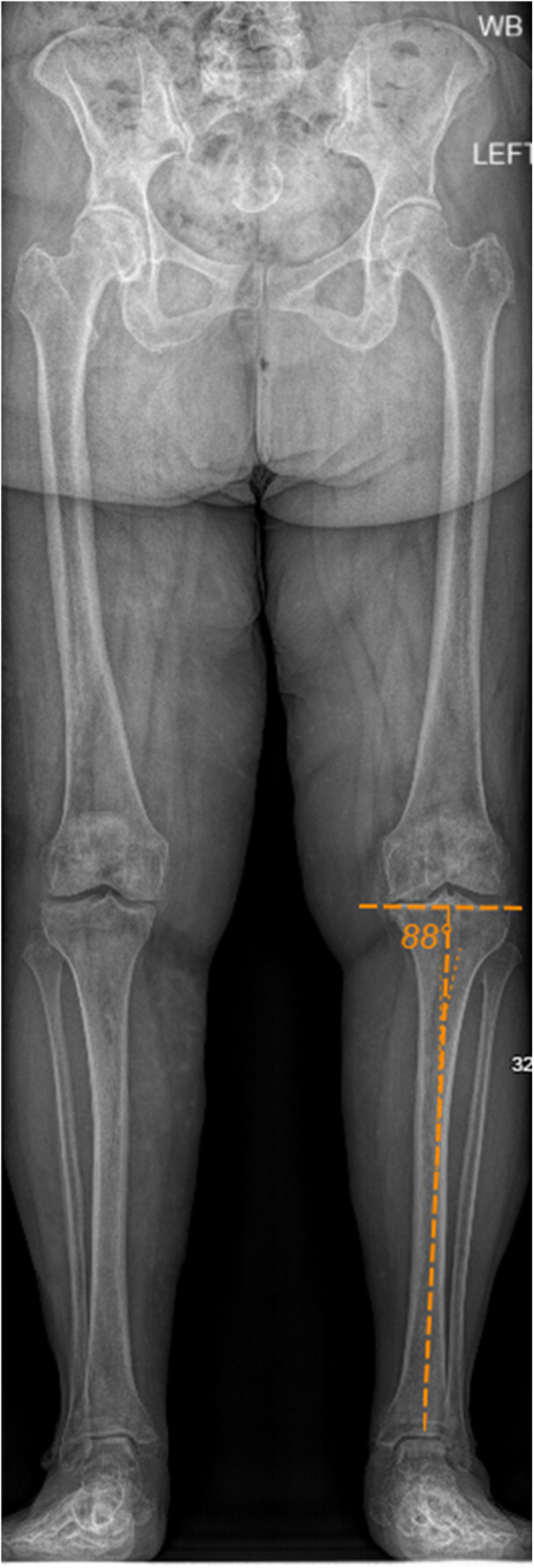
Preoperative long leg views with medial proximal tibial angle measurement.

A contemporary, restricted inverse kinematic TKA technique is described, with osteotomies performed by conventional instrumentation using the *Attune Knee System* (DePuy Orthopaedics). Preoperative computed tomography, navigation or robotic assistance are not required. The tibial osteotomy is restricted to a medial proximal tibial angle (MPTA) of 92–84° in keeping with previously published iKA boundaries which have shown no adverse effects on implant survivorship and represent 93% of native Caucasian MPTAs [1, 18, 23, 40, 45, 48]. Preoperative planning includes MPTA measurement on 3‐foot standing radiographs in all cases (Figure 1) to anticipate the tibial jig setup and requirement for the 2° cutting block described below. Though intraoperative measurements guide resections, standard anterior/posterior (A/P) and lateral radiographs demonstrate tibial slope, posterior offset and patellofemoral height. While note is made of medial and lateral wear patterns, the mHKA is not measured. The technique is applicable to the classically described varus, neutral or valgus alignments.

This technique is applicable to primary cruciate retaining (CR) and posterior stabilised (PS) TKAs; with fixed bearing or rotating platform designs, and for both cemented and cementless techniques.

## TECHNICAL NOTE

After anaesthesia and supine positioning with typical supporting bolsters, a chlorhexidine wash and sterile drapes are applied. A tourniquet is not used for cementless prostheses, and a standard medial parapatellar approach is performed without a medial release. The infrapatellar fat pad is partially resected followed by anterior cruciate ligament resection.

**Figure 2:**
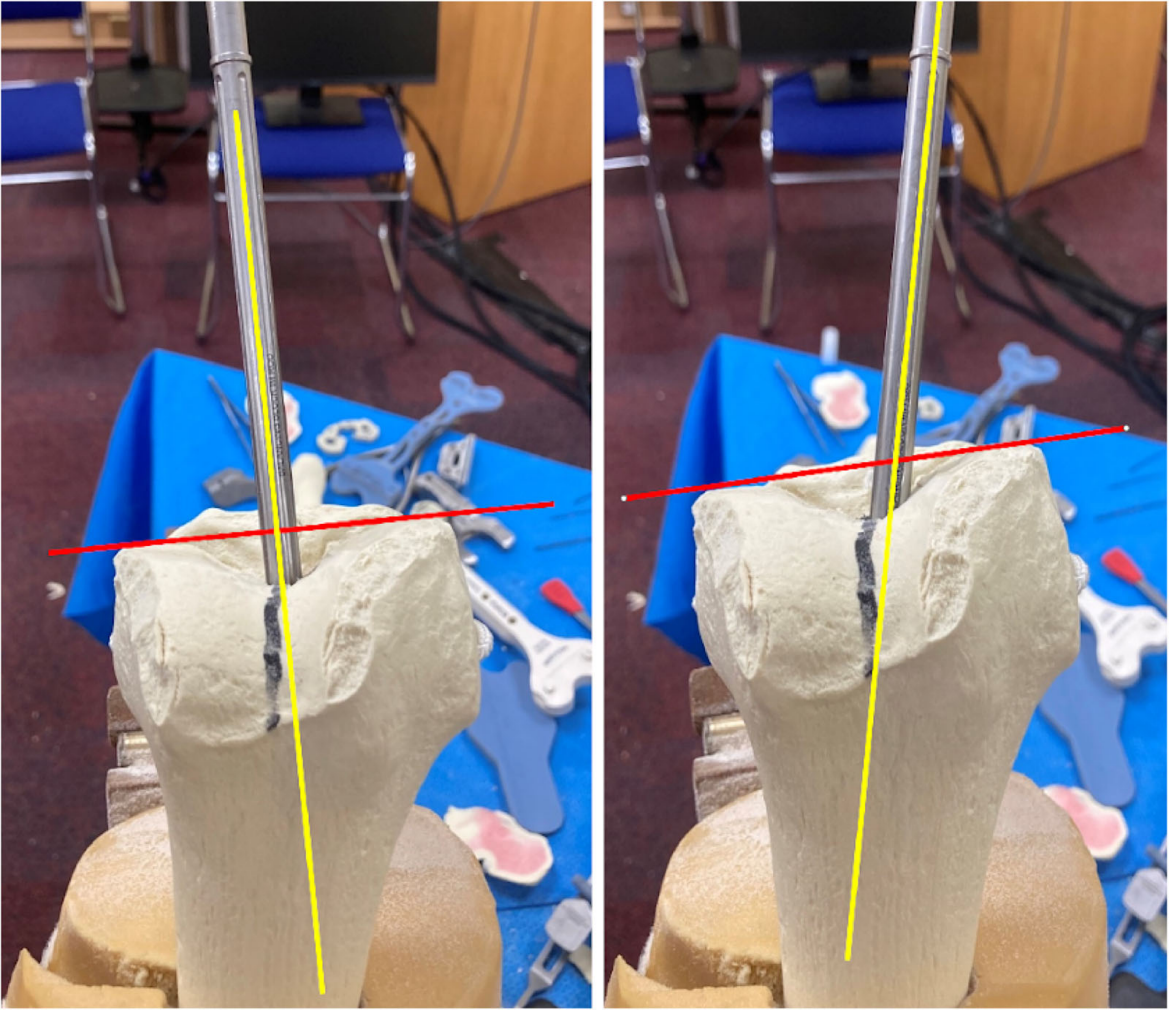
Demonstration of unreliability of intramedullary rod placement for lateral distal femoral angle measurement, with 15° variation easily achievable.

**Figure 3:**
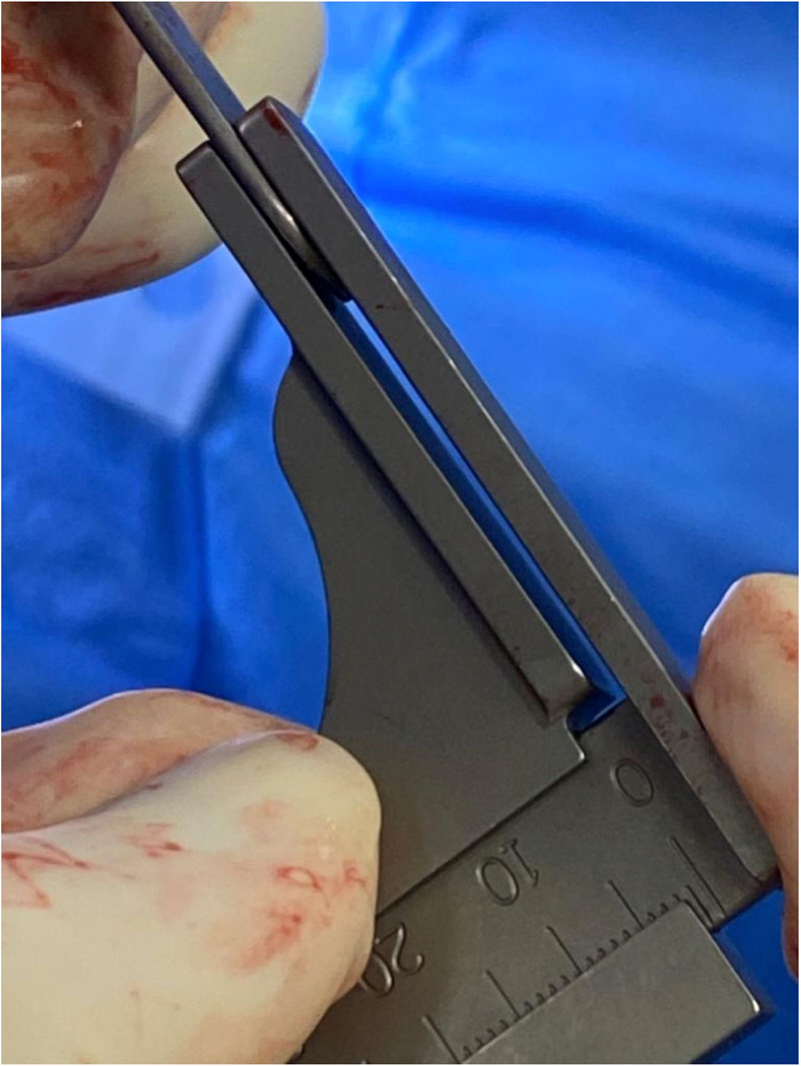
2 mm scalpel handle.

**Figure 4:**
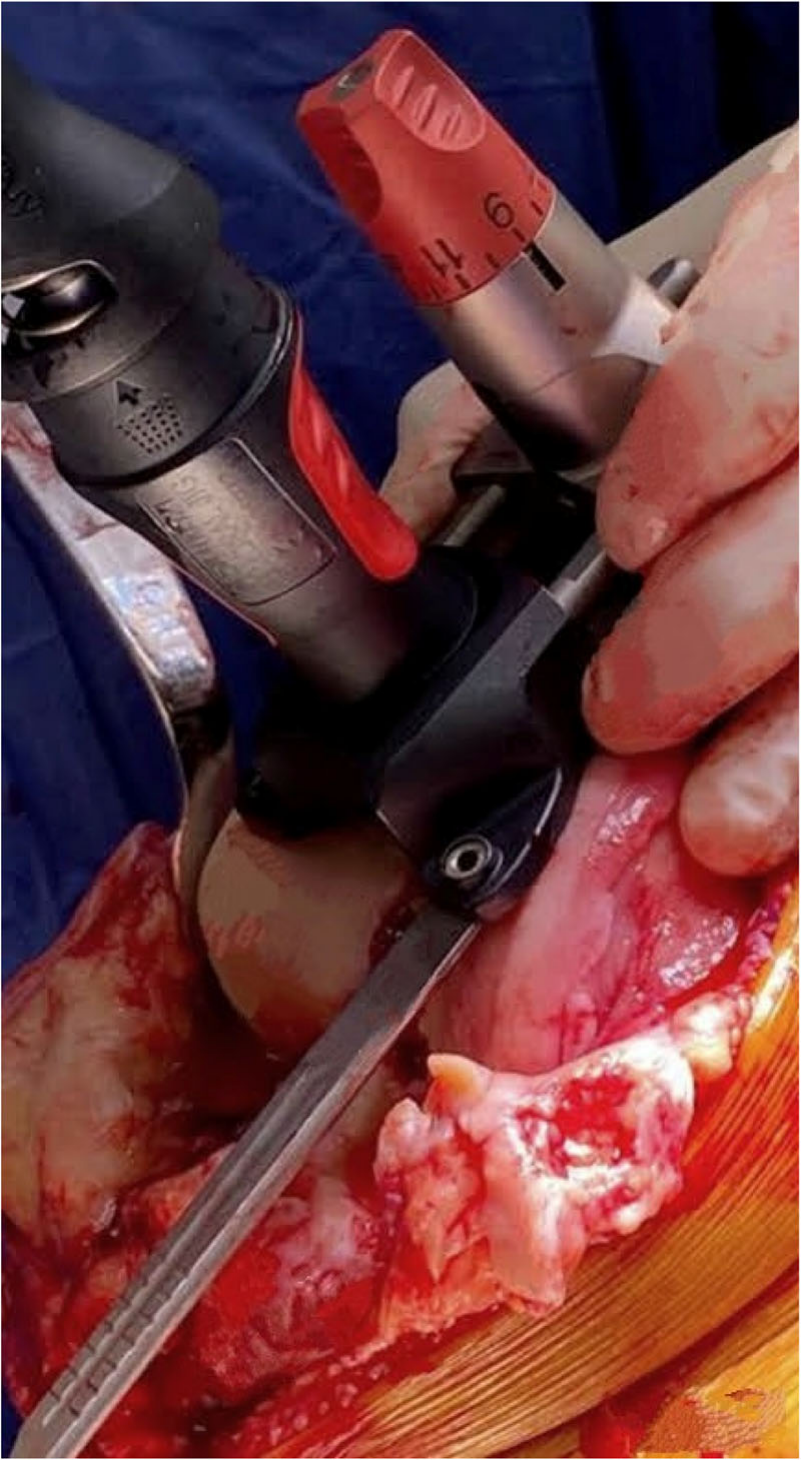
Use of a 2 mm scalpel handle as an aid to achieving a kinematic conservative distal femoral resection.

**Figure 5:**
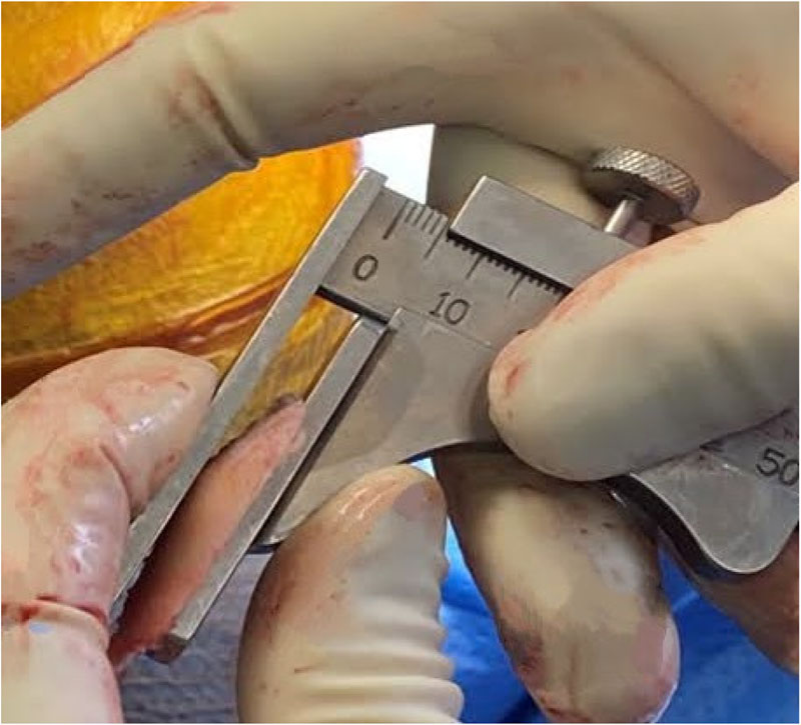
Using a calliper to confirm medial and lateral distal femoral condyle resection heights.

**Figure 6:**
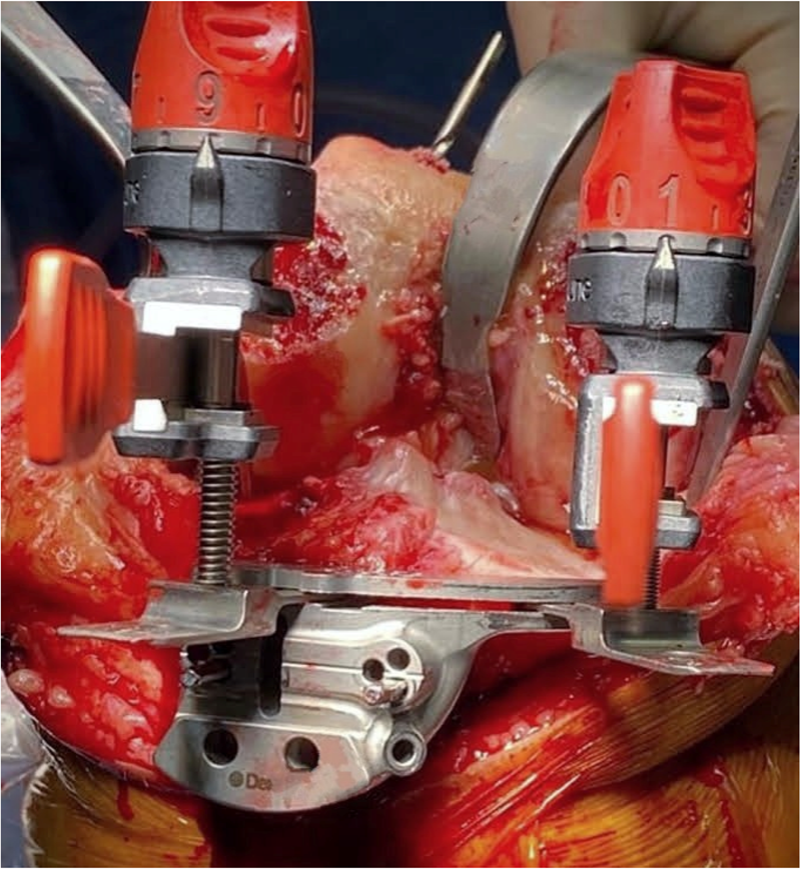
Application of the double‐stylus technique for tibial resection.

**Figure 7:**
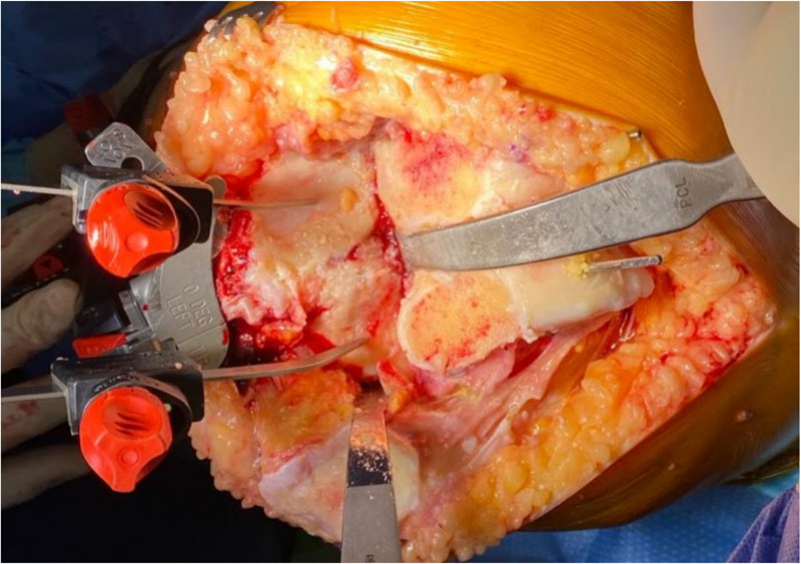
Double stylus placement bird's eye view.

**Figure 8:**
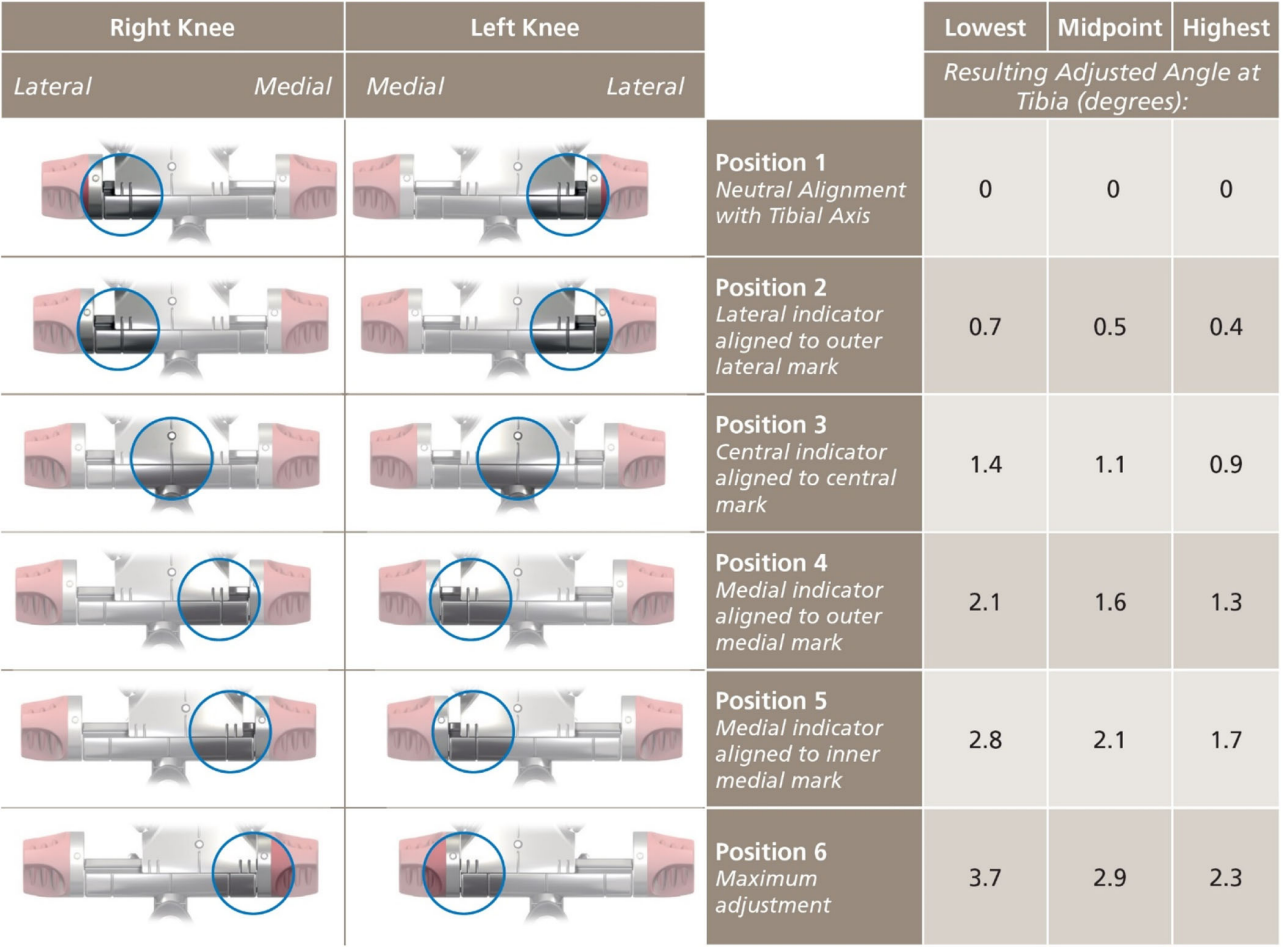
Tibial jig adjustment with corresponding planned medial proximal tibial angle.

**Figure 9:**
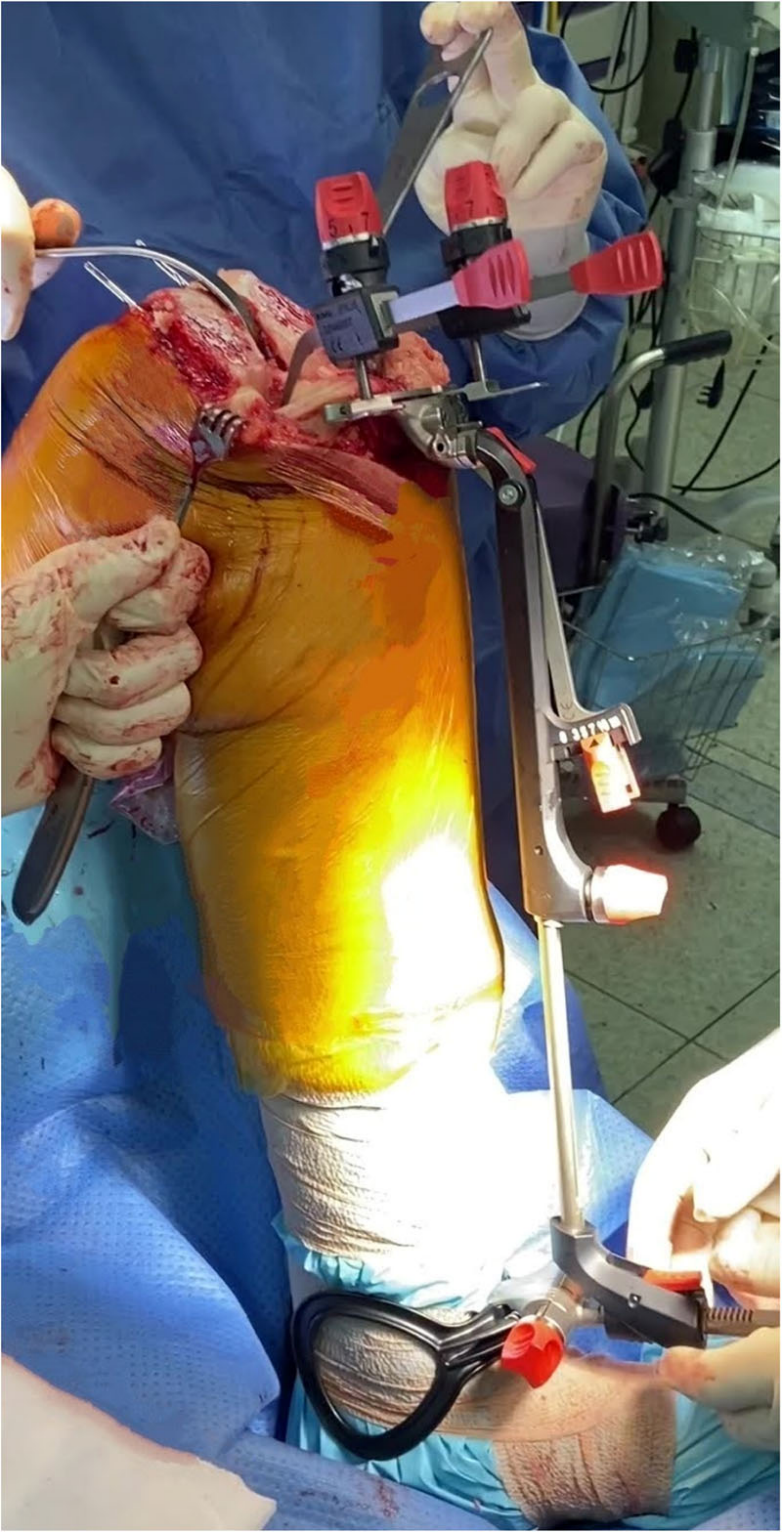
Tibial resection setup using both the double stylus technique and the extramedullary alignment jig.

**Figure 10:**
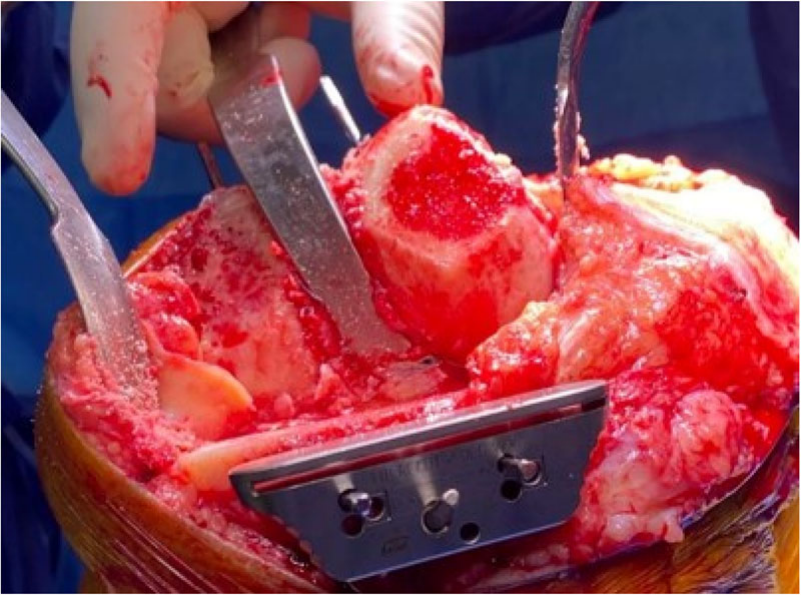
Application of the 2° varus tibial cutting block.

**Figure 11:**
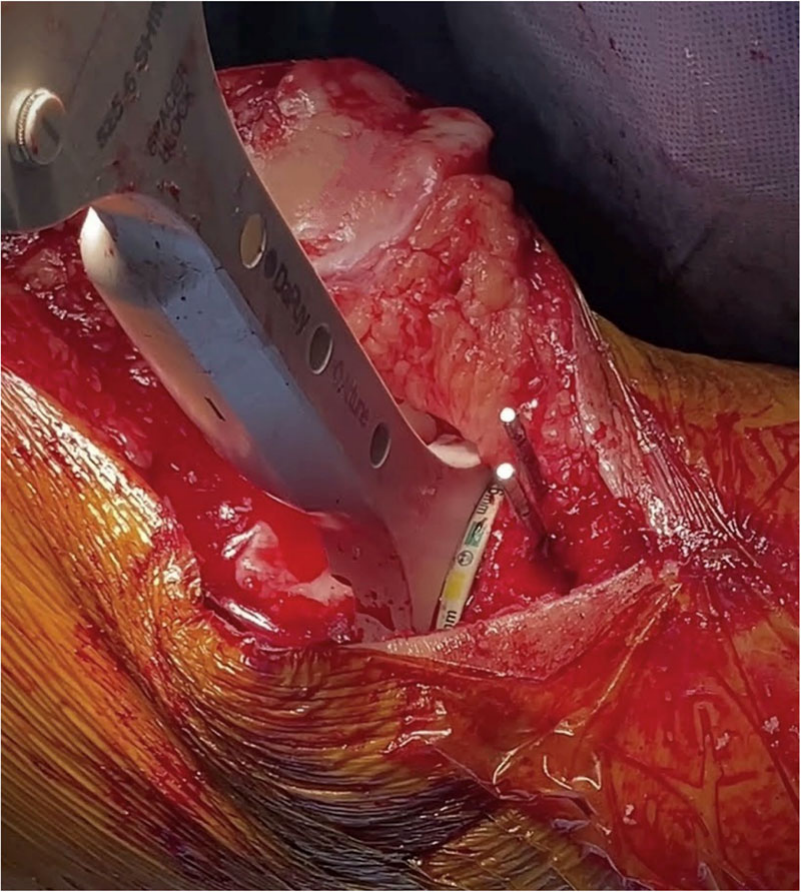
Conservative extension gap balancing.

**Figure 12:**
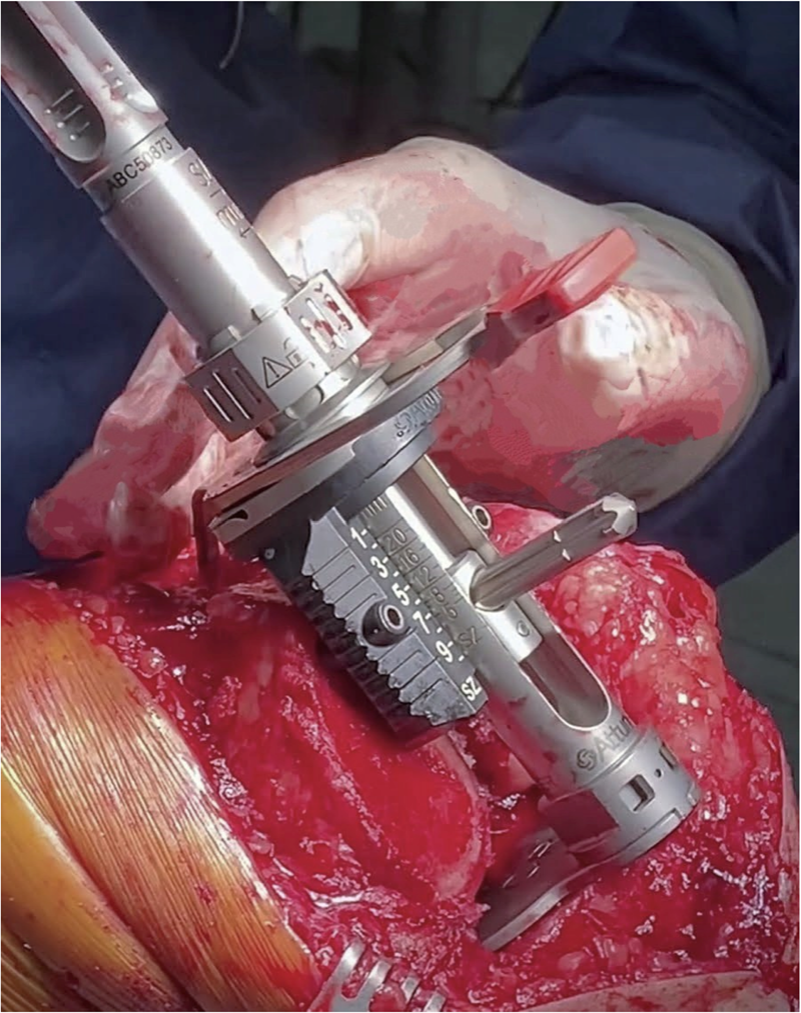
Femoral component sizing and initial component rotation setting.

**Figure 13:**
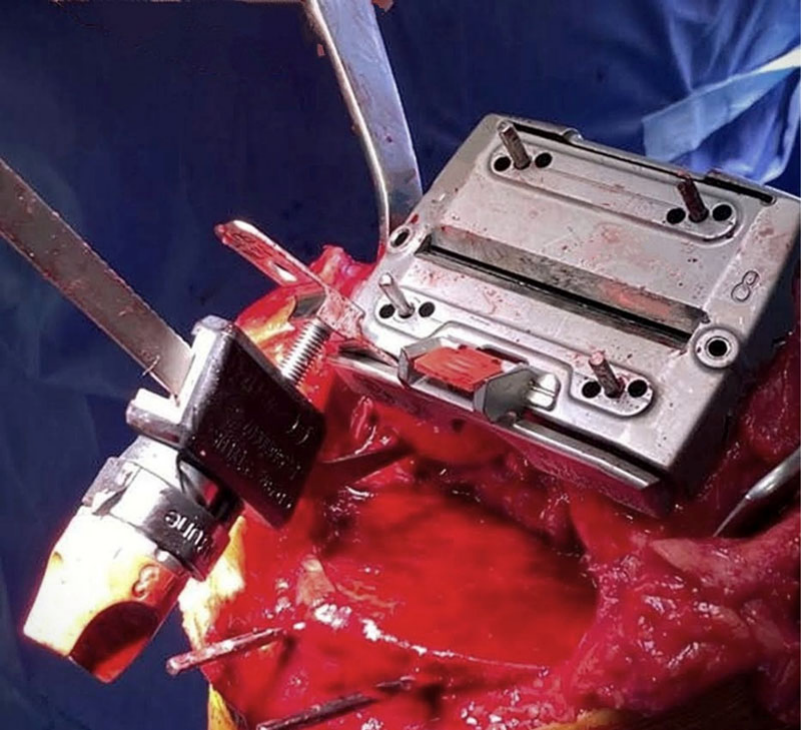
Kinematic posterior condylar resection planning.

**Figure 14:**
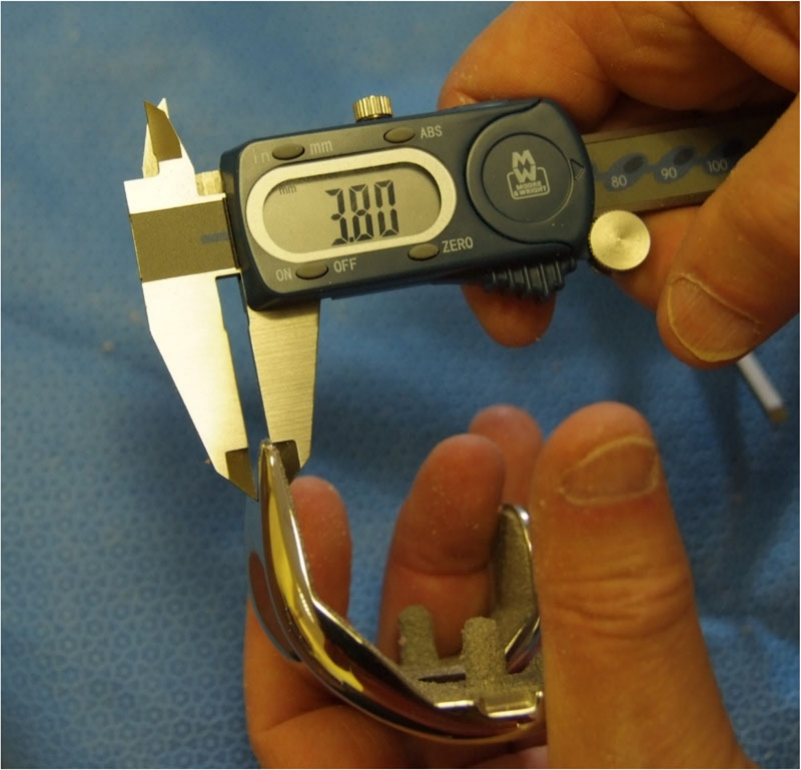
Kinematic restoration of patellofemoral compartment.

**Figure 15:**
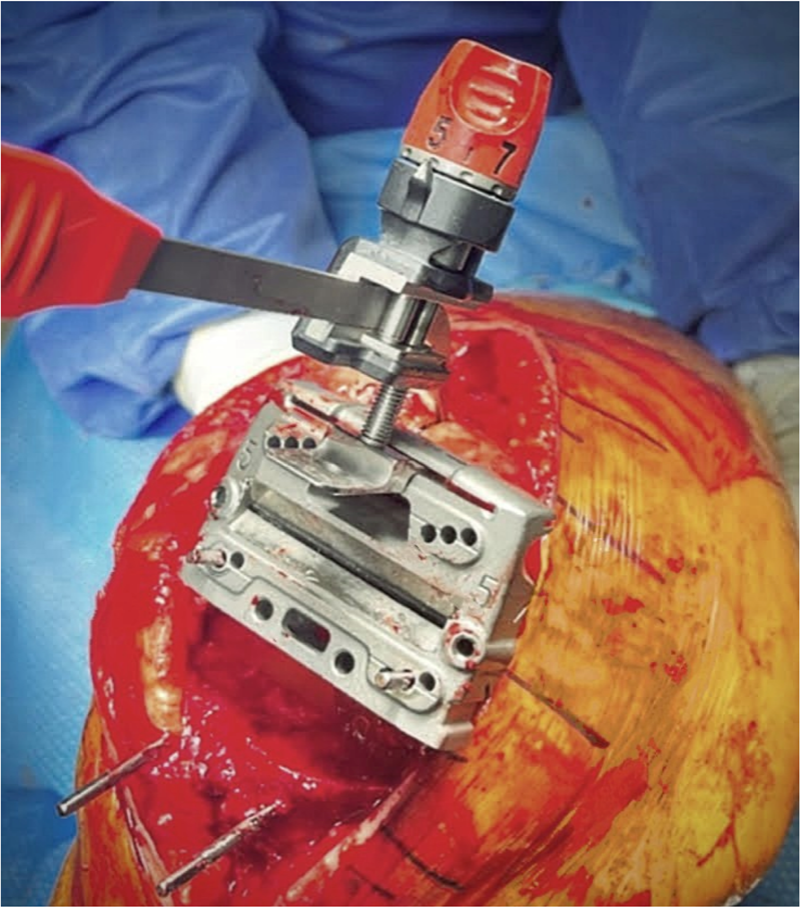
Kinematic restoration of the trochlea.

**Figure 16:**
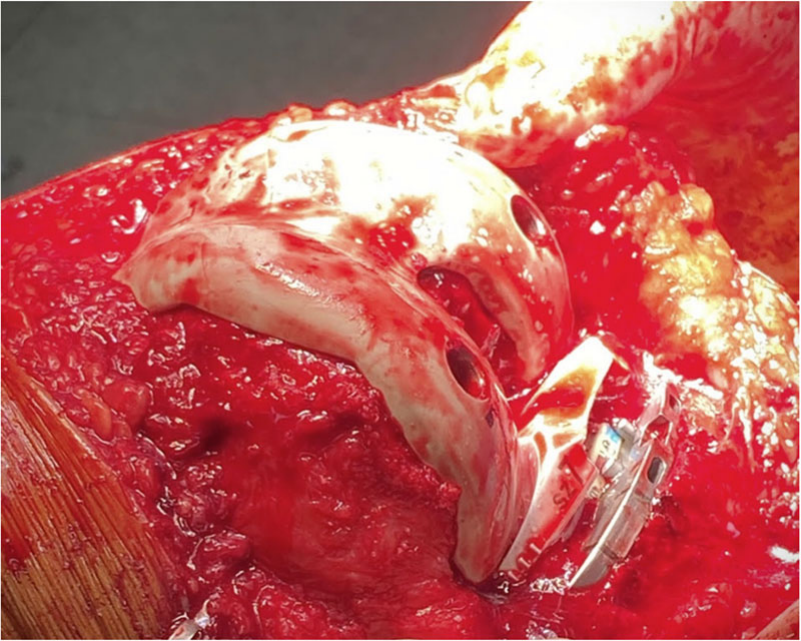
Trial components demonstrating accuracy of femoral cuts.

**Figure 17:**
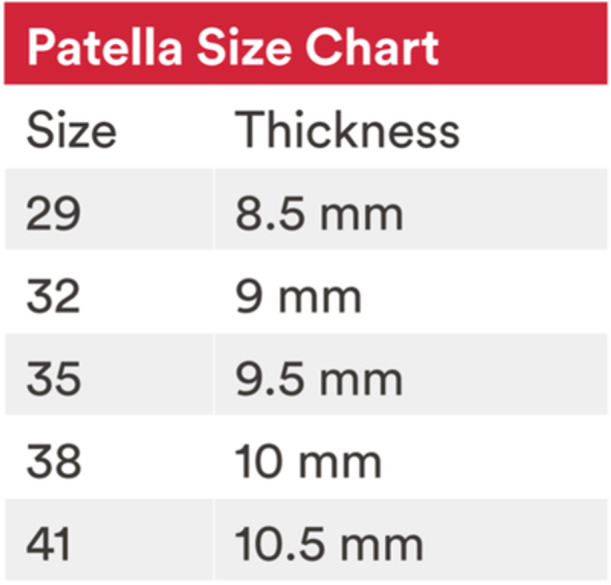
Patellar implant height.

**Figure 18:**
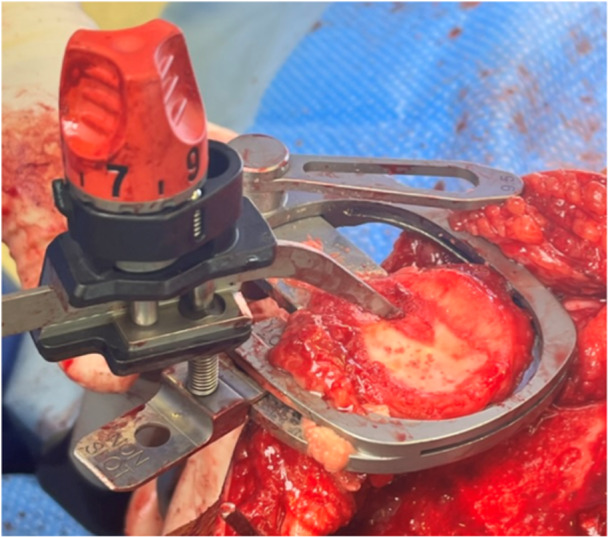
Kinematic restoration of the patella.

**Figure 19:**
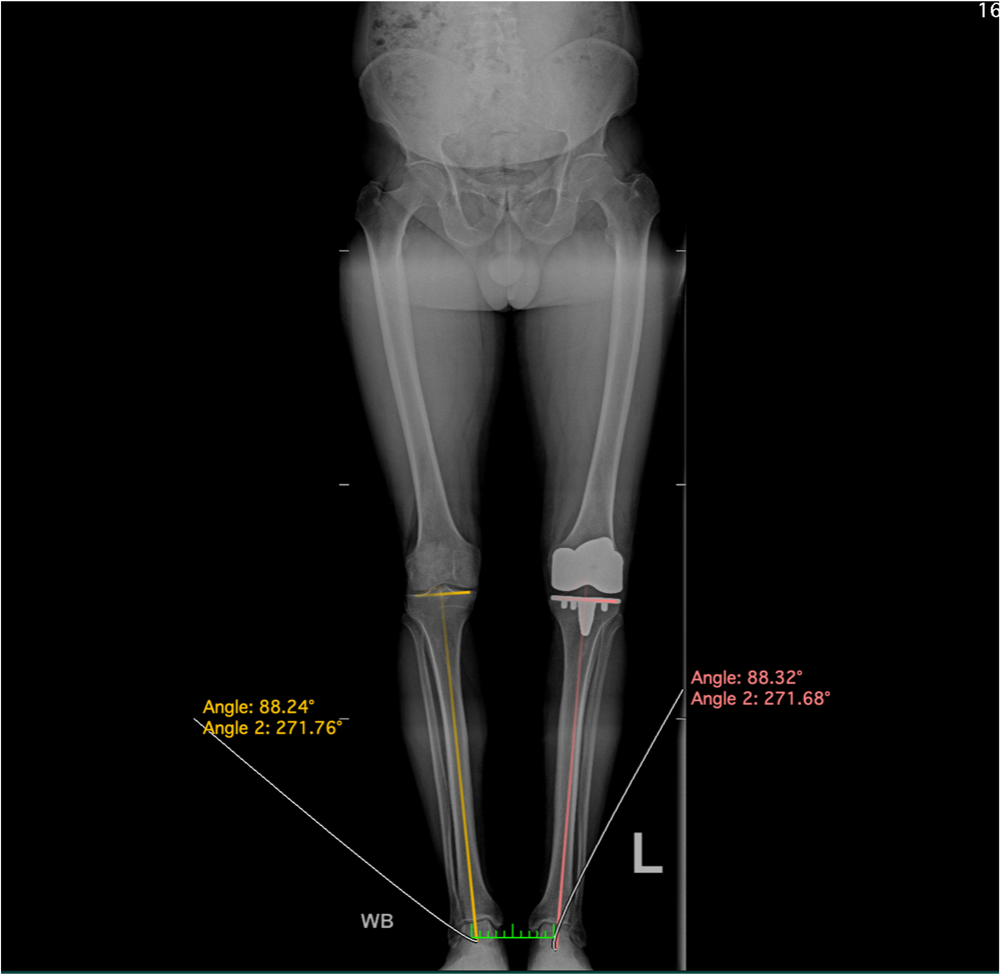
Postoperative radiograph demonstrating restoration of constitutional lower limb alignment, CPAK phenotype and medial proximal tibial angle.

1. *A 5 mm conservative distal femoral resection* is first performed, which will later be followed by an additional, definitive resection to accommodate the 9 mm prosthesis.
  The intramedullary jig‐guided distal femoral cutting block is pinned in place. As the position of the intramedullary rod is an unreliable marker of the femoral axes (Figure 2), the cutting block is positioned on the jig by adjusting the Varus Valgus Dial so that a measured 5 mm medial and lateral distal resection is performed, similarly to KA techniques. The final dial position is noted for later. Note is taken of cartilage loss, with grade 4 wear [27] estimated to be 2 mm depth and so a 3 mm bony resection is performed in the presence of a bald condyle. A scalpel may be used to incise worn cartilage to estimate loss. A standard scalpel handle measured at 2 mm may be placed between a bald condyle and the block (Figures 3 and 4). The medial and lateral distal condylar resections are then measured with a calliper to confirm accuracy (Figure 5). Accounting for 1.19 mm saw blade thickness, and for the observed cartilage loss, a sum total of 5 mm from each side should be noted. If more or less than 5 mm is inadvertently resected, then the later definitive resection is to be altered, as detailed below. The anterior cutting block pins are retained for a later definitive distal femoral resection.
2. *A definitive proximal tibial resection* is performed following anterior tibial dislocation. After removal of large anterior tibial osteophytes, the extramedullary jig allows for positioning of the cutting block as follows:
  a. Axial plane alignment: The block is positioned at the tibial tubercle. To allow for resection height adjustment, the first positioning pin is placed in the central, dynamic slot following the Akagi Line to set axial (rotational) alignment [37].
  b. Resection height: Using a double‐stylus technique (Figure 6), with the tips of the styluses at the peak or trough of the plateaus, usually two‐thirds posterior (Figures 7), 9 mm is resected from both medial and lateral sides. The styluses are placed simultaneously to the below coronal plane setting technique, and in this way, confirm both resection height and coronal plane alignment. Again, 2 mm is allowed for grade 4 wear so that a medial arthritic pattern, for example, would measure 9 mm laterally and 7 mm medially, thereby confirming both resection thickness and patient‐specific coronal plane alignment.
  c. Coronal plane alignment: As the dynamic pin fixation slot used above allows for coronal adjustment, the varus–valgus position of the jig is adjusted with Figure 8 in mind (Figure 9), so that the native MPTA is preserved, as measured on the preoperative radiographs.
    For varus knees with a native MPTA beyond the jig capabilities (which varies with tibial length; Figure 8), the standard cutting block may be temporarily placed before later switching, over the same pins, to the 2° varus cutting block (Figure 10) to perform an additional varus resection.
  d. For CR and PS knees, the resection slope is targeted to 3° or 7°, respectively. To achieve this, a 0° slope cutting block is used, ensuring the jig is parallel to the long axis of the tibia in the sagittal plane and the Posterior Slope Adjustment Lever is set to the above target slope. As with the femoral resections, the resected tibial plateau is measured and confirmed to be 9 mm total from each side, allowing for cartilage (and bony) loss and saw blade thickness.
    As a variation, Step 2 may be performed before Step 1: the tibial resection may be first performed, before the conservative distal femoral resection is performed in the manner described.
3. *The extension gap balance is assessed* after resection of the menisci, ACL and all medial, lateral and posterior osteophytes that may tension the collaterals or posterior capsule (Figure 11). A 14 mm spacer block accounts for the 5 mm conservative distal femoral cut and the 9 mm definitive tibial resections that have been performed (Table 1).
  Where both balance and femoral resection heights are satisfactory, the retained distal femoral pins may be used to perform a parallel 4 mm definitive distal femoral resection. As the −4 mm holes were first pinned for the conservative resection, the 0 mm holes are now used for the same cutting block.
  When balance adjustments are required, this is performed by adjusting the planned definitive distal femoral resection (tibial resections have been confirmed using the double stylus technique, MPTA measurement and calliper checks). If required (approximately 10%–15% of cases), in keeping with iKA principles, the extension gap is balanced by the definitive distal femoral resection. No soft tissue releases are required. To achieve this, the distal femoral pins are removed and the intramedullary jig is reinserted. By adjusting the Varus Valgus Dial from the previously noted conservative resection position, 4 mm is resected from the correct condyle. For an under‐resected condyle, the jig floats 1 or 2 mm distally, as required, to achieve a total of 9 mm to accommodate the femoral component thickness.
  The extension gap is then reassessed using the 18 mm spacer block where a balanced gap is confirmed (Table 1). Insert thickness may be increased if necessary, however, balance has invariably already been achieved.
4. *Flexion gap balance is achieved*, using the Attune Balanced Sizer (Figure 12). The intramedullary rod is inserted and the femoral component size is measured by anterior stylus referencing. Using the same instrument, tibial referenced femoral component rotation is set to match the extension gap insert thickness previously measured. In order to ensure a true kinematic flexion gap, a stylus is used to measure an 8 mm (*Attune* CR thickness) or 9 mm (*Attune* PS thickness) posterior resection from both condyles, again after accounting for wear (Figure 13).
  The A/P Chamfer Block is placed and further stabilised by four more pins to ensure perfect resections for the cementless component. Anterior, posterior and chamfer resections are performed, ensuring to clear all debris from the posterior knee. The femoral cut assessment tool confirms precise resections.
5. *Kinematic patellofemoral replacement is planned*: For the *Attune* system, the trochlear height of all femoral components sizes is 3.8 mm (Figure 14). During step 4, after placement of the 4‐in‐1 cutting block, a stylus is used to measure the planned anterior femoral cut with the tip of the stylus at the midpoint of the trochlea (Figure 15).
  To prevent over or under‐stuffing (notching) of the third compartment, the femoral component may be up or downsized at this point, without altering the flexion or extension gaps. To achieve this, the posterior pins are retained while the two cross pins and 2 anterior pins are removed and the block is exchanged in size over the posterior pins to target a 3.8 mm trochlear resection (or 1.8 mm if again accounting for 2 mm cartilage loss), thereby restoring the normal trochlear joint line. Although a satisfactory trochlear resection height may be measured, care should be taken to avoid anterior femoral notching, especially if downsizing. An angel wing may be used for assurance.
6. *The sulcus or notch resections* are performed using the CR or PS guides, respectively.
7. *Trial components are inserted* and alignment, range, stability, balance, tibial rotation and patellar tracking are assessed. For the cementless technique, particular attention is paid to the accuracy of cuts (Figure 16).
8. *Patellar resurfacing* begins by using a calliper to ensure that 12 mm of bone remains after the planned cut. The Patellar Resection Guide and stylus are placed, with the stylus set on the highest point of the patella. The stylus is set to resect a height matching the planned *Attune Medialised Anatomic Patella implant* height (Figure 17), with again 2 mm allowed for full‐thickness cartilage loss (Figure 18). Care must be taken to ensure the plane of resection is parallel to the coronal plane of the patella and so patellar tilting is avoided. A trial patellar component confirms kinematic restoration and normal tracking of the patellofemoral joint before final implants and cement are requested.
9. *Final femoral and tibial preparations* for the lugs, keel and pegs are performed. Time allows for local anaesthetic to be injected before host surfaces are washed and dried.
10. *The components are implanted*, with intraarticular tranexamic acid injected after capsular closure in flexion. Standard skin closure and dressing application are performed. Drains or circumferential dressings are not required. Postoperative long‐leg radiographs confirm the restoration of constitutional coronal alignment and JLO (Figure 19).

**Table 1:** Gap checking explained.

	Planning for	Target	Created by	Spacer block
Conservative extension gap check	N/A	14 mm	5 mm conservative distal femoral resection 9 mm tibial resection	5 mm shim 9 mm spacer block
Definitive extension gap check	9 mm femoral component 5 mm polyethylene insert 4 mm tibial tray	18 mm	5 mm conservative distal femoral resection 4 mm definitive distal femoral resection 9 mm tibial resection	5 mm shim 13 mm spacer block
Or same table transposed, per copyeditor's preference		

	Conservative extension gap check	Definitive extension gap check
Planning for	N/A	9 mm femoral component 5 mm polyethylene insert 4 mm tibial tray
Target	14 mm	18 mm
Created by	5 mm conservative distal femoral resection 9 mm tibial resection	5 mm conservative distal femoral resection 4 mm definitive distal femoral resection 9 mm tibial resection
Spacer block	5 mm shim 9 mm spacer block	5 mm shim 13 mm spacer block

## DISCUSSION

This surgical technique paper details an instrumented TKA technique that is founded upon the principles of both KA and iKA. Those familiar with the *LCS Complete Knee System* (DePuy Orthopaedics) may identify with many of the principles described. Practicing and aspiring arthroplasty surgeons pursuing a robust, reproducible technique that avoids the higher costs, resource commitments, complexities and potentially less favourable revision rates of technology‐assisted TKA techniques may wish to consider the described procedure [22, 43, 51]. In addition, kinematic alignment strategies likely achieve higher patient satisfaction rates [10, 38, 47, 49].

This technique incorporates a Sequential Engineering process, whereby each step is carried out separately and the next step cannot begin until checks have been completed [35]. For example, resection thicknesses are confirmed by calliper technique before proceeding. In addition, engineered redundancy assures extremely accurate cuts and reduces error. For example, both the double stylus technique and the MPTA measurement technique ensure kinematic tibial alignment and the flexion gap balancer combined with the posterior condylar measurement confirms a kinematic posterior femoral resection.

While the described technique utilises the principles of both KA and iKA, it is considered that both the femoral and tibial final resections are independently kinematically performed. When no alignment alterations are required via definitive distal femoral resection adjustments and a parallel further 4 mm osteotomy is performed, the joint has undergone independent femoral and tibial kinematic resections. The conservative extension gap check merely ensures that the distal femoral and tibial resections, performed independently of each other, achieve a balanced extension gap. In addition, precise, measured resections of the anterior femur and patella allow for kinematic restoration of the third compartment of the knee.

Surgeons may be enticed to consider the conservative distal femoral resection a redundant or expendable step. However, the authors find the incidence of alignment adjustments via the final definitive osteotomy to be frequent. In perhaps 10%–15% of cases, subtle coronal plane adjustments are performed by altering the definitive distal femoral resection to achieve perfect final extension gap balance. Proceeding directly to a definitive distal femoral resection by skipping a conservative resection would risk a less accurately balanced extension gap and therefore risk: raising the joint line by requiring further femoral resections, altering the JLO through tibial resections or otherwise unnecessary defunctioning soft tissue restraints. Surgeons may, however, choose the variation described in Step 2 above where the tibial resection is first performed, depending on the preference.

Technical considerations for this technique include the necessity to ensure femoral and tibial resections of known heights. The definitive distal femoral resection is planned from the conservative extension gap testing using a 14 mm spacer block to account a 9 mm tibial cut (and 5 mm femoral cut). However, if more or less than 9 mm has been resected from the tibia, the spacer block must be altered accordingly. For example, following an inadvertent 11 mm tibial resection, an 11 mm block should be used in order to avoid under‐resection of the definitive distal femoral resection and subsequent distalisation of the joint line. Less than 9 mm tibial resections require further revision tibial resections to achieve the minimum tibial component height. The described double‐stylus technique followed by secondary checks by calliper measurement of the resected plateau offer reassuring accuracy and consistency.

Limitations of this technique include a literature paucity concerning the long‐term outcomes of iKA using conventional instrumentation. Parente et al. have reported no difference in patellofemoral outcomes comparing conventionally instrumented iKA to MA [33].

Second, as tibial bone loss is more commonly encountered than femoral, the native joint line may be more difficult to estimate using iKA compared to KA in such cases. The authors find that tibial bone loss is usually posteromedially located and the stylus finds the native joint line more anterior to the deficit. In cases of severe bone loss, the unworn anterior and central tibial plateau may be extrapolated to estimate the native joint line. However, tibial restoration in complex primary procedures with the most severe tibial bone loss are not within the remit of this article.

In addition, this technique utilises the *Attune* system incorporating the *Attune Gradius* radius design. Previous studies have described the benefits of a single radius design during kinematic femoral techniques in order to replicate the Cylindrical Axis of the Knee. However, femoral roll‐back in flexion may be lost with the resulting increased deep flexion constraint. Multiradius designs have sought to address this, but flexion instability and paradoxical anterior tibial translation have been cited as causes of concern with previous ‘J‐curve’ designs [13]. The *Attune Gradius* curve claims to balance the concerns of both single and multiradius designs by providing a gradual and much lesser overall reduction in posterior radius [8]. However, further studies are required to specifically compare long‐term design outcomes.

Further limitations include the exclusion of both complex primary and revision TKA procedures, implant survivorship, functional outcomes; radiographic outcomes; PROMS and comparative outcomes, all of which will be separately reported.

The authors find the described technique to be uncomplicated, reproducible and robust. In addition, the utility of this technique for CR or PS; fixed or mobile bearing; cemented or cementless designs allows for the application of the technique according to surgeon preference and patient characteristics. For surgeons interested in pursuing the benefits of a patient‐specific, instrumented technique for TKA, the described strategy and surgical technique is recommended.

## AUTHOR CONTRIBUTIONS

All authors contributed significantly to this technique paper.

## CONFLICT OF INTEREST STATEMENT

The authors declare no conflict of interest.

## ETHICS STATEMENT

The authors have nothing to report.

## Supporting information

Supporting information.
