# Supplementary material for: Conventionally instrumented inverse kinematic alignment for total knee arthroplasty: How is it done?
Source: J Exp Orthop. 2024 Jun 12;11(3):e12055. doi: 10.1002/jeo2.12055 (PMC11167404; doi:10.1002/jeo2.12055)

Appendix 1

For varus arthritic knees with severe medial wear, that may include tibial bone loss, an inadequate tibial resection may be seen (Figure A). In such cases, bony sclerosis may be observed following the initial cut (Figure B). A 2 degree cutting block may be used to further resect the medial tibial plateau to achieve a resection height equal to the component height of 9mm in total (Figure C).


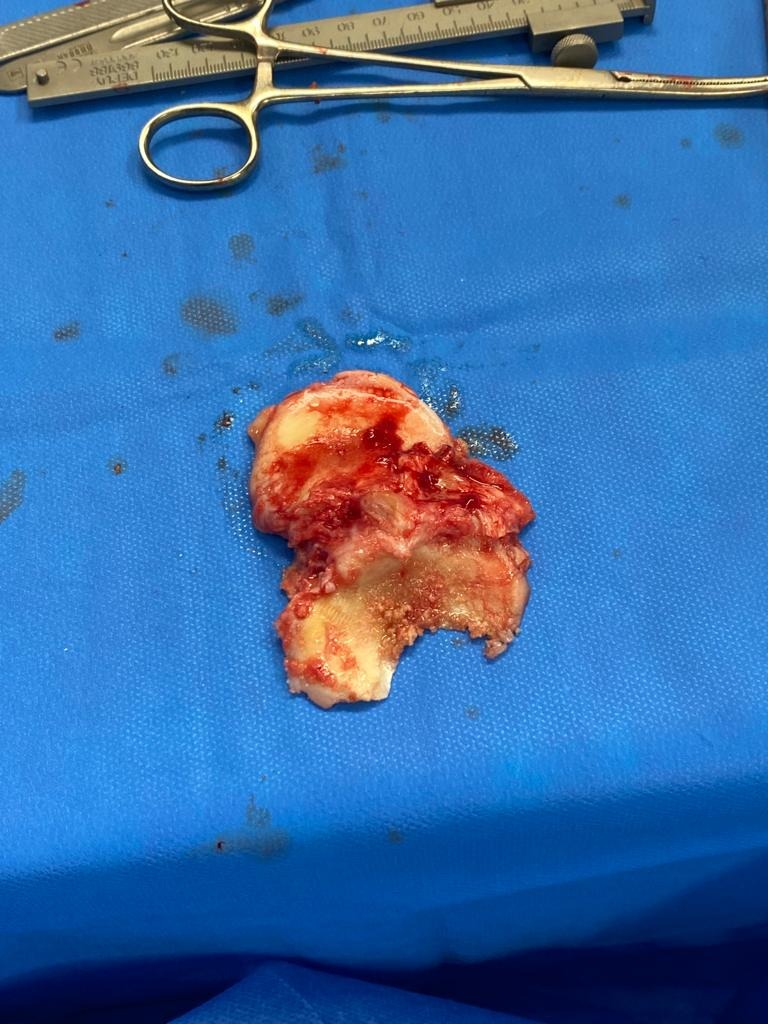


Figure A: Inadequate tibial resection


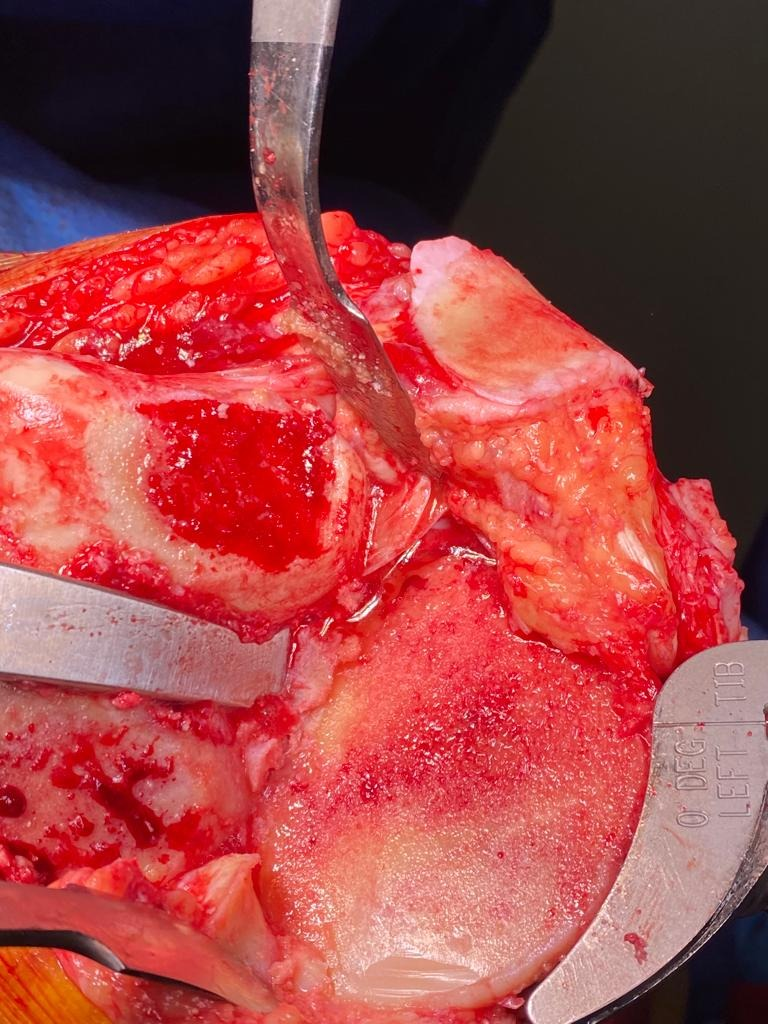


Figure B: Sclerotic medial tibial plateau


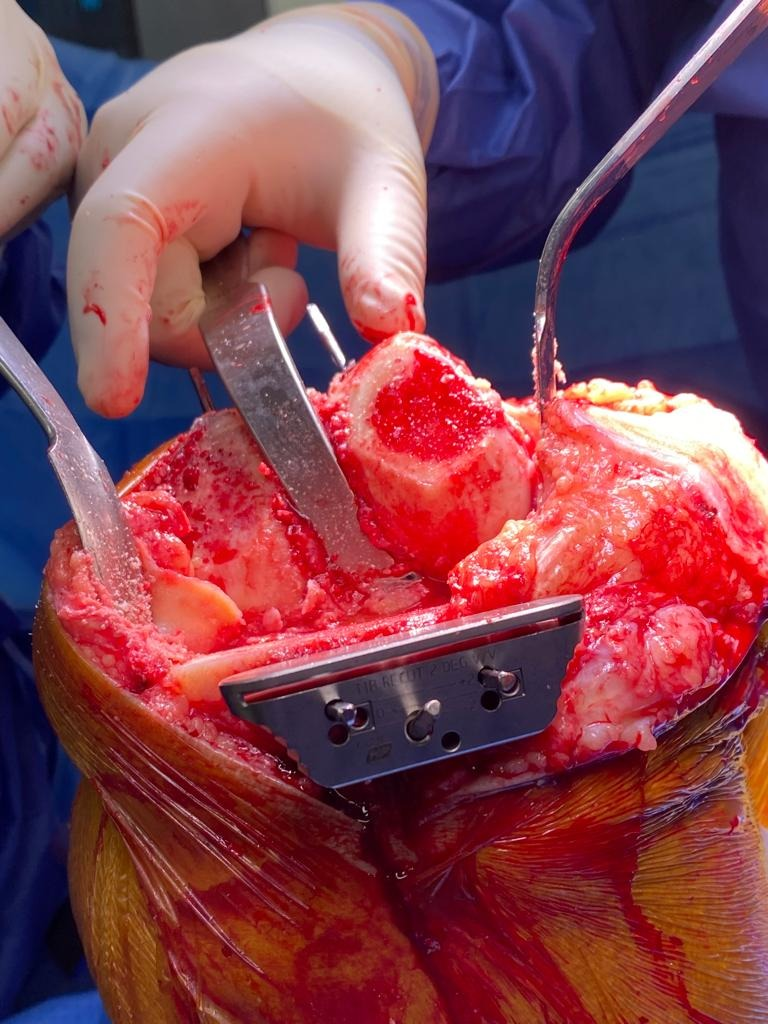


Figure C: Use of a 2 degree cutting block to achieve the targeted tibial resection height


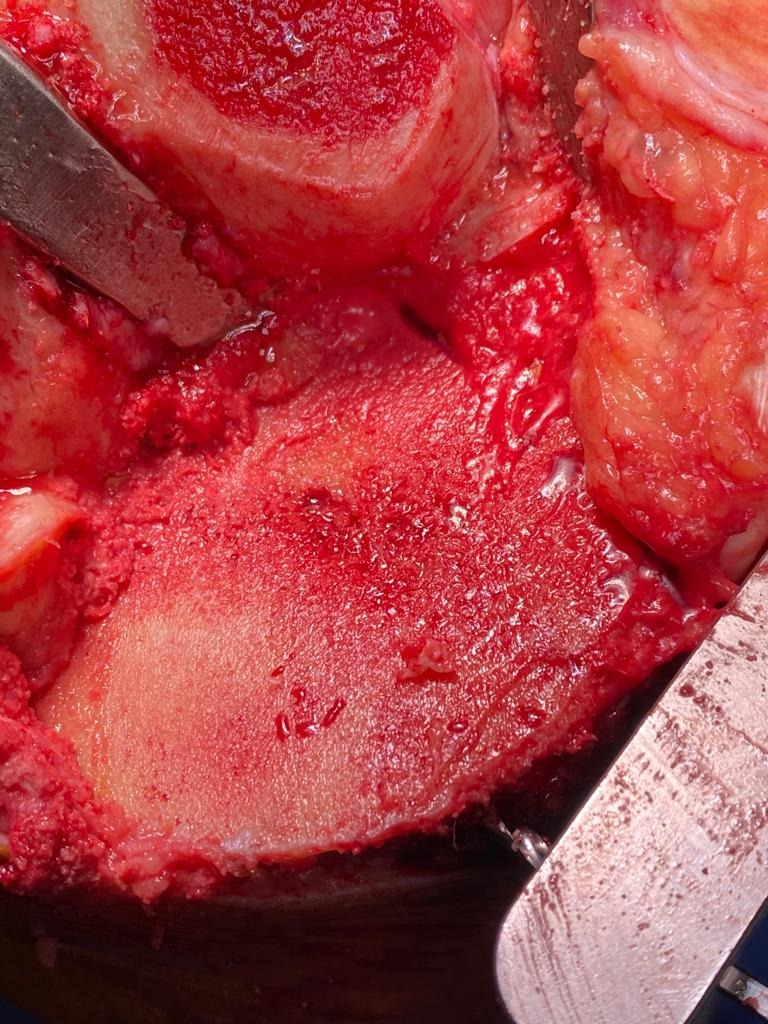

Supplement: Supplementary file 1 — Supporting information. [file JEO2-11-e12055-s001.docx]
